# Intrauterine Intussusception Causing Ileal Atresia

**Published:** 2010-12-01

**Authors:** Yogesh Kumar Sarin

**Affiliations:** Department of Pediatric Surgery, Maulana Azad Medical College New Delhi, India

**Keywords:** Intrauterine Intussusception, Jejuno-ileal atresia, Neonatal intestinal obstruction, Etiology

## Abstract

Intrauterine intussusception (IUI) is the one of the rarest recognized causes of jejuno-ileal atresia (JIA). We report on a 15-day old full-term neonate presenting with features of intestinal obstruction, wherein on exploration, a visible ileo-ileal intussusception resulting in ileal atresia was found. The relevant literature has been reviewed.

## INTRODUCTION

IUI has been described as a rare cause of intestinal atresia. Evans, in a review of almost 1500 cases of intestinal atresias found only 9 (0.6%) cases due to IUI. However, the pathophysiology of this condition remains unclear because the diagnosis is usually made postnatally based on the intra-operative findings of neonatal surgery [1,2].

IUI as cause of JIA was first recognized by Chiari in 1888, and then reported for the first time in English literature by Davis and Poynter in 1922. In 1983, Pavri et al could find less than 30 adequately documented cases of JIA resulting from IUI. An extensive review of world literature revealed less than 100 cases of this association till date; only 2 cases reported from India hitherto. Only isolated case reports are available; though two series from Japan have reported more than 10 cases of JIA resulting from IUI in the last decade [3-9].

In this report a full-term neonate who was diagnosed to have JIA resulting from IUI intra-operatively, is reported.

## CASE REPORT

A male neonate, born to a 19-year-old primigravida at full term, of a consanguineous marriage, through normal vaginal delivery at home by a traditional birth attendant, presented at 15th day of life with bilious vomiting, failure to pass meconium and progressive abdominal distension since birth. The mother had not received any antenatal care. The baby was being breast fed all this while and he had only received some intravenous fluids for few hours before admission. His general condition was poor. A clinical diagnosis of intestinal obstruction was made, and confirmed by plain abdominal radiographs (Fig. 1).

**Figure:**
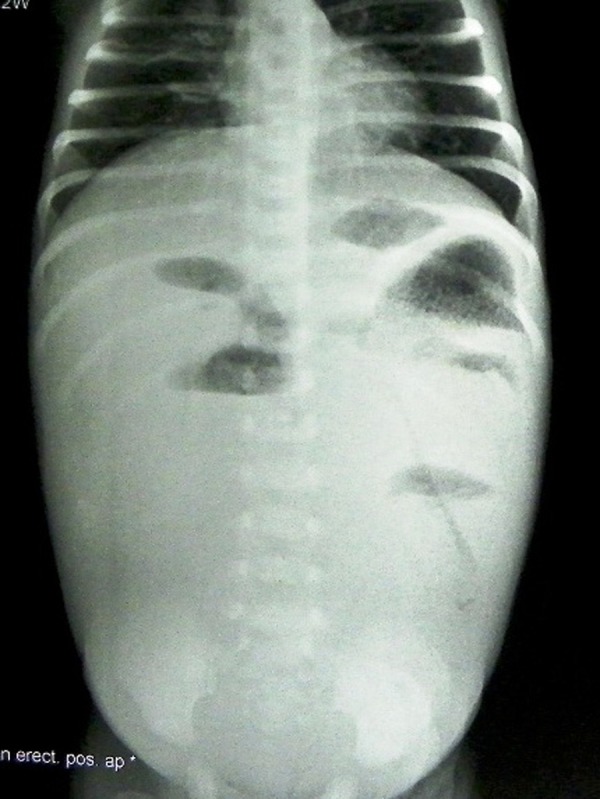
Figure 1: Plain X-ray abdomen shows distal bowel obstruction.

After adequate resuscitation, laparotomy was undertaken that revealed adhesions and calcareous deposits suggestive of meconium peritonitis and type IIIa ileal atresia, 15cm proximal to ileocecal valve. The distal collapsed ileal segment revealed an obvious intussusception (Fig. 2); a 5 cm long viable ileal segment could be retrogradely reduced from this segment of ileum. No pathologic lesion as lead point could be identified. An 8 cm of dilated proximal and 6cm of the distal ileum was resected and end- to-back anastomosis was performed. Histopathology of the resected specimen showed transmural inflammation of both proximal and distal bowel segments. The postoperative period remained stormy including sepsis, disseminated intravascular coagulation and meningitis. The child however survived and discharged on 17th postoperative day.

**Figure:**
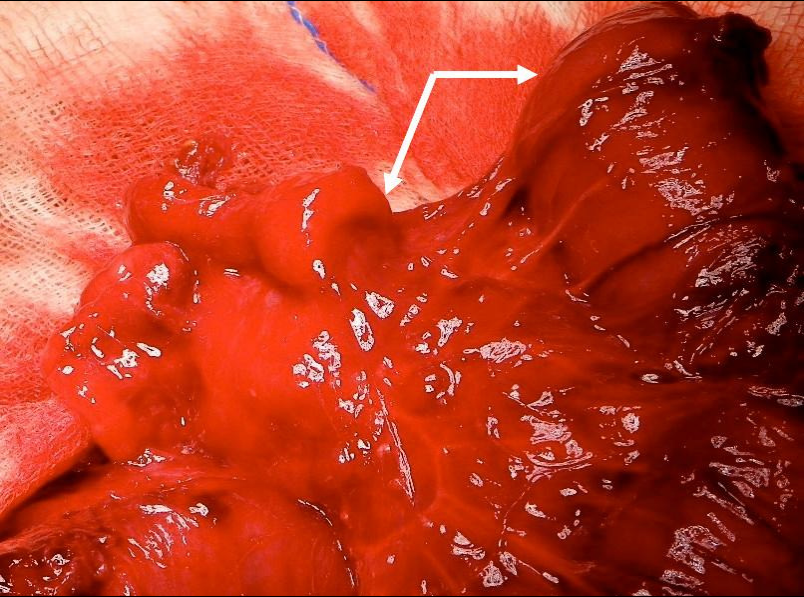
Figure 2: Intussuscepted bowel seen at the distal segment of ileal atresia. The two white thick arrows point at the two ends of the ileal atresia.

## DISCUSSION

Mid and low JIA is generally considered to result from intrauterine vascular disruption(s) in a part of the developed intestine occurring relatively late in gestation. These disruptive intrauterine events may include volvulus, herniation, constriction, thrombosis of mesenteric vessels and rarely intussusception. At least on one occasion, both intrauterine volvulus and IUI have been concomitantly noted in the same neonate with JIA. No local vascular disruptive event, however, is known to occur in patients with apple-peel atresia (APA) or multiple atresias (MA). Genetic, placental, or other causes have been speculated for APA and MA. The exact incidence of IUI is not known. The underlying IUI has been noted in 0.6 to 25% cases of JIA reported previously [1,9,10].

IUI has rarely been detected by prenatal ultrasonography after 25-30 weeks of pregnancy. Isolated or transient fetal ascites has been suggested as a pointer to IUI. Other sonographic features described include dilated intestinal loops with high echogenicity and a "target-like" appearance. Abdominal calcifications may also be seen. Though there are no reports of fetal therapy once an antenatal diagnosis of IUI has been made, but an early diagnosis would definitely result in referral to a tertiary neonatal surgical center and early surgical intervention for better outcome [2,11,12]. Although survival in the present case is encouraging, but non-availability of antenatal care and approaching medical facility so late could only be condemned.

IUI causing JIA is usually seen in full-term neonates but has been reported rarely in prematurely born neonates. The condition is considered to occur very late in pregnancy, which is supported by presence of normal or near-normal sized colon due to passage of meconium in majority of these neonates and absence of associated anomalies [9,10,13].

A definitive preoperative diagnosis may be difficult, if not impossible. Though the usual presentation is that of neonatal intestinal obstruction, and features of peritonitis are usually absent, dilated proximal bowel may perforate and lead to pneumoperitoneum. The diagnosis is usually made at surgery or more commonly at histopathological examination [7,14].

JIA due to IUI is single, usually of types II and IIIa, and of mid and low jejuno-ileum. IUI has never been encountered along with high jejunal, APA or MA. Pathologically, the lesion is identical to classic, idiopathic intussusceptions as seen in infants and older children; the presence of lead point such as Meckel’s diverticulum has been reported rarely [7,9,10,12].

IUI and resultant JIA was readily evident as in our case and in few instances before, so a cause-effect relationship could be established, but this may not always be the case. In those situations, IUI may be revealed only on histopathological examination. On histology, usually a polypoid intussusceptum, with relatively good preservation of the structure of the intestine, is observed at the obstructed end on the distal side. In few cases with the markedly necrotic tissue, polypoid lesion was found located apart from the blind end. Rarely, intussusceptum and intussuscipiens have been seen to be fused [6-8].

The present case reaffirms that IUI may occur at late stage of pregnancy and cause impairment of blood supply to a segment of intestine leading to its resorption and atresia.

## Footnotes

**Source of Support:** Nil

**Conflict of Interest:** None declared
